# Dysbiotic oral microbiota contributes to alveolar bone loss associated with obesity in mice

**DOI:** 10.1590/1678-7757-2022-0238

**Published:** 2022-11-21

**Authors:** Ian de Meira Chaves, Marina Campos Zicker, Alice de Oliveira Laranjeira, Ana Letícia Malheiros Silveira, Daniele Cristina de Aguiar, Breno Rocha Barrioni, Adaliene Versiani de Matos Ferreira, Mauro Martins Teixeira, Tarcília Aparecida da Silva, Daniele da Glória de Souza, Mila Fernandes Moreira Madeira

**Affiliations:** 1 Universidade Federal de Minas Gerais Instituto de Ciências Biológicas Departamento de Microbiologia Belo Horizonte MG Brasil Universidade Federal de Minas Gerais, Instituto de Ciências Biológicas, Departamento de Microbiologia, Belo Horizonte, MG, Brasil.; 2 Universidade Federal de Minas Gerais Escola de Enfermagem Departamento de Nutrição Belo Horizonte MG Brasil Universidade Federal de Minas Gerais, Escola de Enfermagem, Departamento de Nutrição, Belo Horizonte, MG, Brasil.; 3 Universidade Federal de Minas Gerais Instituto de Ciências Biológicas Departamento de Bioquímica e Imunologia Belo Horizonte MG Brasil Universidade Federal de Minas Gerais, Instituto de Ciências Biológicas, Departamento de Bioquímica e Imunologia, Belo Horizonte, MG, Brasil.; 4 Universidade Federal de Minas Gerais Instituto de Ciências Biológicas Departamento de Fisiologia e Farmacologia Belo Horizonte MG Brasil Universidade Federal de Minas Gerais, Instituto de Ciências Biológicas, Departamento de Fisiologia e Farmacologia, Belo Horizonte, MG, Brasil.; 5 Universidade Federal de Minas Gerais Departamento de Engenharia de Metalúrgica e de Materiais Belo Horizonte MG Brasil Universidade Federal de Minas Gerais, Departamento de Engenharia de Metalúrgica e de Materiais, Belo Horizonte, MG, Brasil.; 6 Universidade Federal de Minas Gerais Faculdade de Odontologia Departamento de Clínica, Patologia e Cirurgia Odontológicas Belo Horizonte MG Brasil Universidade Federal de Minas Gerais, Faculdade de Odontologia, Departamento de Clínica, Patologia e Cirurgia Odontológicas, Belo Horizonte, MG, Brasil.

**Keywords:** Periodontal diseases, Obesity, Alveolar bone loss, Oral microbiota, Mice

## Abstract

Periodontal diseases (PD) are inflammatory conditions that affect the teeth supporting tissues. Increased body fat tissues may contribute to activation of the systemic inflammatory response, leading to comorbidities. Some studies have shown that individuals with obesity present higher incidence of PD than eutrophics.

**Objective:**

To investigate the impact of obesity on periodontal tissues and oral microbiota in mice.

**Methodology:**

Two obesity mice models were performed, one using 12 weeks of the dietary protocol with a high-fat (HF) diet in C57BL/6 mice and the other using leptin receptor-deficient mice (*db/db-/-*), which became spontaneously obese. After euthanasia, a DNA-DNA hybridization technique was employed to evaluate the microbiota composition and topical application of chlorhexidine (CHX), an antiseptic, was used to investigate the impact of the oral microbiota on the alveolar bone regarding obesity.

**Results:**

Increased adipose tissue may induce alveolar bone loss, neutrophil recruitment, and changes in the oral biofilm, similar to that observed in an experimental model of PD. Topical application of CHX impaired bone changes.

**Conclusion:**

Obesity may induce changes in the oral microbiota and neutrophil recruitment, which are associated with alveolar bone loss.

## Introduction

Periodontal diseases (PD) are inflammatory conditions characterized by damage of the teeth supporting tissues. The dysbiosis of the oral microbiota is a determining factor in the pathogenesis of PD.^1^ *Aggregatibacter actinomycetemcomitans* (*Aa*), a Gram-negative bacterium, is an important microorganism related to PD, acting as a key participant in oral dysbiosis.^2^

Studies also show that systemic conditions, such as obesity, diabetes and rheumatoid arthritis impact PD.^3,4,5^ Thus, patients with obesity, for example, are at a higher risk of developing PD.^3^

Obesity is an abnormal or excessive fat accumulation that can impair health.^6^ White adipose tissue is an endocrine organ related to the synthesis of compounds such as adipocytokines, associated to energy homeostasis^7^ and to immune response regulation.^8^ Diverse evidence supports endocrine regulation of bone metabolism by adipose tissue.^9^ In mice, Montalvany-Antonucci, et al.^10^(2018) showed harmful effects of HF diet on bone microarchitecture.^10^ However, the mechanisms that underlie the association between PD and obesity are deficiently understood.

Obesity causes dysbiosis and, on the contrary, a dysbiotic microbiota may favor obesity.^11,12^ In a dysbiotic context, some microorganisms can interfere in the physiological functions of the immune system, and, in an oral environment it may contribute to PD progression.^1^ Obesity may affect periodontal tissues by causing an exacerbated systemic inflammatory response.^13^ Our hypothesis is that the systemic inflammation due to obesity induces oral dysbiosis, which contribute to alveolar bone loss. Therefore, we evaluated the oral microbiota in two models of obesity in mice and whether dysbiosis contributed to alveolar bone loss observed in obese mice.

## Methodology

### Mice

C57BL6/J wild-type (WT), specific pathogen-free mice, were obtained from the animal facility of the Federal University of Minas Gerais (Universidade Federal de Minas Gerais, UFMG, Brazil), and leptin-receptor deficient mice (*db/db*^-/-^) were obtained from the Immunopharmacology laboratory facilities (UFMG, Brazil). The mice were housed in separate cages, under standard conditions and with free access to food and water. All animals were from 6 to 8 weeks old and were separated according to gender. Depending on the experiment, 4, 5, or 10 mice were randomly allocated to each group. Sample size was decided by power analysis. The experimental protocol was approved by the local Institutional Animal Ethics Committee (CEUA/UFMG) under protocol 272/2014, in accordance with the ARRIVE guidelines.

### Diet

WT mice were separated in two groups: i) fed with standard laboratory chow (Labina Essence^®^, Brazil) or ii) fed with a 45% high-fat (HF) diet. Composition of the control chow (Labina) was 60% carbohydrate, 11% fat and 23% protein. The HF diet was prepared by an alteration in standard AIN-93M,^14^ modified to achieve 45% fat calories. After 12 weeks of dietary protocol, the mice were euthanized and samples of maxilla, jaw, blood, Epididymal Adipose Tissue (EAT), Retroperitoneal Adipose Tissue (RAT), Inguinal Adipose Tissue (IAT), and Mesenteric Adipose Tissue (MAT) were collected. The adiposity index was calculated using the visceral adipose tissue weight, following the formula: [(EAT + RAT + MAT) / body weight in grams] × 100.^15^ All samples were evaluated in a blinded manner by a single examiner (MCZ).

### Periodontal infection

For this experiment, WT mice were divided in 4 groups: i) control or sham-infected mice under standard diet; ii) control or sham-infected mice under HF diet; iii) infected mice under standard diet; iv) infected mice under HF diet. After 8 weeks under the standard or HF diet, two groups of WT mice received oral inoculation of *A. actinomycetemcomitans* ATCC 43718, from collection of the Microorganism-Host Interaction Laboratory (UFMG). The bacteria were cultured in Tryptic Soy Agar (TSA, Bhaveshwar Plaza, Lbs Marg-Mumbai, India) plus 0.5% of yeast extract (Oxoid^®^ Basingstoke, Hampshire, UK) at 37°C and in a microaerophilic atmosphere using a glass jar, in a Biochemical Oxygen Demand (BOD) incubator (Thermo Scientific, Waltham, MA, USA), for 48 h. Subsequently, the bacteria were cultured in Tryptic Soy Broth — (TSB - Oxoid^®^, Hampshire, UK), plus 0.5% of yeast extract (Oxoid^®^), and maintained for 24 h at 37°C in a microaerophilic atmosphere. A medium containing *A. actinomycetemcomitans* was centrifuged (3.075 g for 5 min, at room temperature) and the pellet was suspended in Phosphate-Buffered Saline (PBS) to obtain an inoculum with 1 × 10^9^ CFU/mL. For PD induction, as described by Madeira, et al.^16^ (2012), 100 μL of inoculum plus 1.5% carboxymethylcellulose was placed in the mice's oral cavity, using a micropipette. The sham-infected mice were administered 100 uL of PBS with 1.5% carboxymethylcellulose in the oral cavity. The protocol was repeated after 48 and 96 h.

### Quantification of alveolar bone loss

The evaluation of alveolar bone loss was performed as previously described.^16^ The maxillae were hemisected, exposed to 15% hydrogen peroxide overnight, and mechanically defleshed. Then, they were stained with 0.3% methylene blue. The palatal faces of the molars were photographed with 20× magnification using a stereomicroscope (Metrimpex Hungary/PZO, Labimex, Hungary) and a digital camera (Kodak EasyShare C743, Rochester, USA). The images were analyzed using the Image J software (National Institutes of Health, USA). Quantitative analysis was used to measure the area between the Cement-Enamel Junction (CEJ) and the Alveolar Bone Crest (ABC) in the first upper right molar. All samples were evaluated in a blinded manner by a single examiner (IMC).

### Myeloperoxidase concentration

The activity of myeloperoxidase (MPO) in the mice's periodontal tissues was measured as previously described.^16^ After euthanasia, hemimaxillae, including teeth, periodontal soft tissues, and alveolar bone were removed and processed. Subsequently, the samples were assayed and MPO activity was measured by changes in Optical Density (OD) at 450 nm, using tetramethylbenzidine (1.6 mM) and H_2_O_2_ (0.5 mM). The results were expressed as MPO activity by 100 mg of tissue.

### Enzyme-Linked Immunosorbent Assay (ELISA)

The adipocytokine concentrations were measured in the mice's maxillae and/or serum. For protein extraction, the palatal periodontal tissue was homogenized in PBS containing anti-proteases (0.1 mmol/L phenylmethylsulfonyl fluoride, 0.1 mmol/L benzethonium chloride, 10 mmol/L EDTA, and 20 KI aprotinin A) and 0.05 % Tween 20, pH 7.4, and the supernatants were stored at −20°C. Cytokines were measured using ELISA kits according to the manufacturer's instructions (R&D Systems, Minneapolis, MN, USA). The results were expressed as cytokine picograms per 100 mg of maxilla tissue or per 1 mL of serum.

### Micro Computed Tomography (Micro-CT)

The mice's maxillae were fixed in 10% neutral buffered formalin for 48 h and scanned using a microCT system (Skyscan 1174 — Bruker-MicroCT, Kontich, Belgium). Calibration was carried out with known-density calcium hydroxyapatite phantoms (Skyscan). High-resolution scans with an isotropic voxel size of 18 μm were acquired (50 kV voltage, 0.5 mm aluminum filter, 0.5° rotation angle). Trabecular morphometry was measured within the furcation area of the first molar root. The tissue was analyzed to determine Bone Mineral Density (BMD g/cm^2^), Bone Volume (BV mm^3^), Percent Bone Volume/Tissue Volume (BV/TV %), trabecular thickness (Tb.Th mm), trabecular number (Tb.N 1/mm), and trabecular separation (Tb.Sp mm) as previously described.^17^ The images were analyzed with the Dataviewer and CTAn software (Bruker-MicroCT). All samples were evaluated in a blinded manner by a single examiner (IMC).

### Bacterial quantification by DNA-DNA hybridization

Oral bacterial quantification was obtained by means of Checkerboard DNA-DNA hybridization. After euthanasia, maxillae samples were collected and immediately stored at −80°C. DNA extraction was performed following the Genomic DNA Purification Kit protocol (Thermo Scientific, Waltham, MA, USA). The samples were homogenized using TE (10 mM Tris-HCl, 1 mM EDTA, pH 7.6) and evaluated individually by means of Checkerboard DNA-DNA hybridization, as previously described.^18^

### Topical application of chlorhexidine

Topical application of 0.12% chlorhexidine (CHX) plus 2% carboxymethylcellulose, was performed in the mice's oral cavity. For this, 50 μL of CHX were applied with a brush in the oral cavity every 48 h for 4 weeks (from week 8 to week 12).

### Statistical analysis

Kolmogorov-Smirnov was used to analyze normal distribution. One-way ANOVA was employed to analyze differences among the groups, followed by a Newman-Keuls post hoc test. When comparing two groups, a Kolmogorov-Smirnov test and an unpaired Student's t-test were performed. Grouped analysis was made by means of Two-way ANOVA followed by Bonferroni. The data were analyzed using GraphPad Prism 8 (GraphPad Inc., San Diego, CA, USA). p-values <0.05 were considered statistically significant.

## Results

### High-fat (HF) diet induces body weight gain, increased adiposity and higher leptin production in mice.

**Figure 1:**
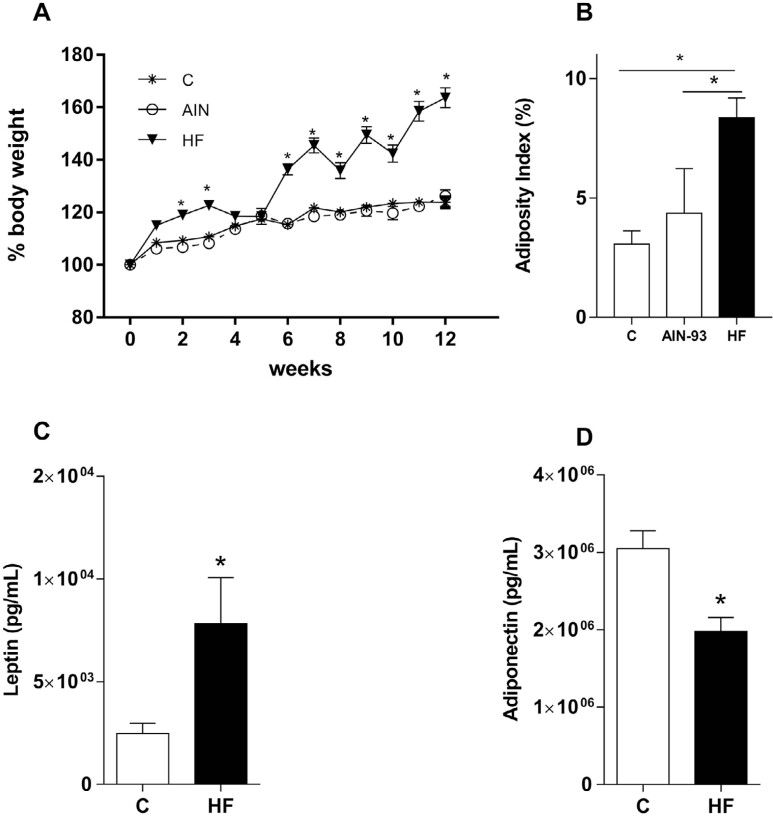
Mice fed HF diet presented body weight gain, increased adiposity, and alteration in the leptin and adiponectin concentrations. A: Weight parameters measured for 12 weeks, once a week, in groups of mice fed with control chow, AIN-93, or HF diet. B: Adiposity index measured in mice fed with control chow, AIN-93, or HF diet after 12 weeks. C: Leptin serum levels after 12 weeks, measured in mice fed with (C) control chow or HF diet. D: Adiponectin serum levels after 12 weeks measured in mice fed with a control diet or HF diet. N=5. The values (mean ± S.E.M) of all experiments were obtained from two independent experiments, with 30 mice. *p<0.05 versus control groups. The grouped analysis was performed by means of two-way ANOVA followed by Bonferroni in the body weight analysis. One-way ANOVA was followed by Newman-Keuls. The Student's t-test was performed when two groups were analyzed

Consumption of a HF diet resulted in increased body weight and adiposity in mice when compared to mice given a control chow purchased from a local supplier — AIN-93M diet (Figures 1A and 1B, respectively), which characterizes the Diet-Induced Obesity (DIO) model. Since the body weight of the control chow remained the same when compared to the AIN-93M chow, the former was chosen as the control diet for further analysis in subsequent experiments. The results also showed increased leptin serum levels (Figure 1C) and decreased adiponectin serum levels (Figure 1D) in mice fed with the HF diet.

### Alveolar bone loss in obese mice

**Figure 2:**
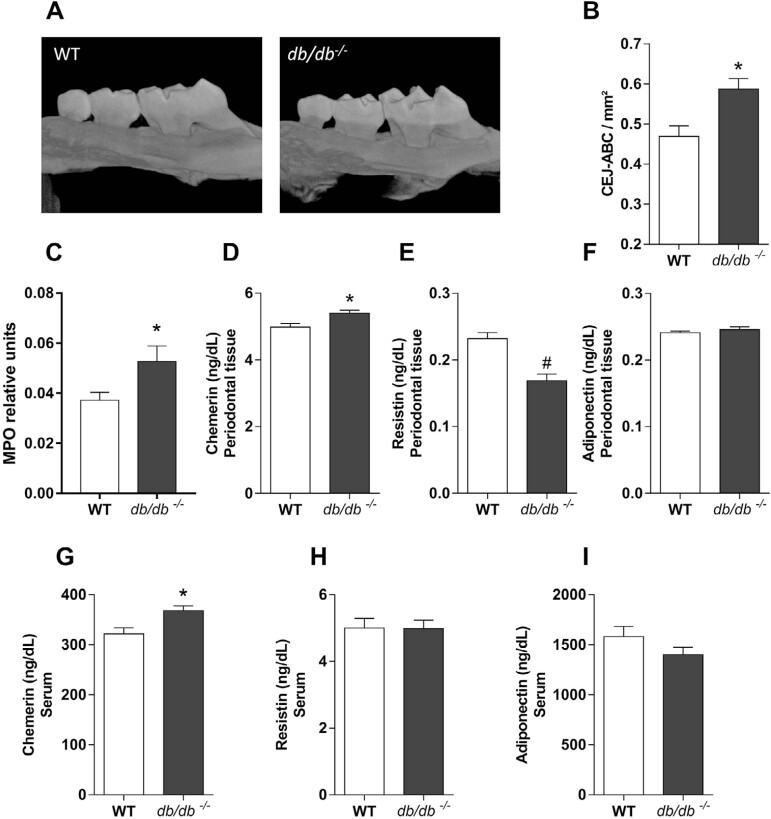
Analysis of periodontal tissues and serum changes in *db/db-/-* mice. (A) Representative images of alveolar bone loss in WT and *db/db-/-* mice. (B) Analysis of alveolar bone loss in WT and *db/db-/-* mice. (C) MPO quantification in periodontal tissues of WT and *db/db-/-* mice. (D) Chemerin concentration in periodontal tissues. (E) Resistin concentration in periodontal tissues; (F) Adiponectin concentration in periodontal tissues; (G) Chemerin serum levels. (H) Resistin serum concentration. (I) Adiponectin serum concentration. N=10, *, #p<0.05 as per Student's t-test. The values (mean ± S.E.M) of all experiments were obtained from two independent experiments, with 40 mice

*db/db*^-/-^ mice have greater changes in metabolic disturbances than WT mice given a HF diet.^19^ In *db/db*^-/-^ mice, we observed increased alveolar bone loss when compared to WT mice (Figures 2A and 2B). Moreover, *db/db*^-/-^ mice showed increased MPO activity (Figure 2C), increased chemerin production (Figure 2D), and decreased resistin concentration in periodontal tissues when compared to WT mice (Figure 2E). No difference was found in the adiponectin levels (Figure 2F). The evaluation of systemic levels of cytokines in *db/db*^-/-^ mice showed increased chemerin serum levels (Figure 2G) but resistin (Figure 2H) and adiponectin (Figure 2I) serum levels remained the same.

### High-fat diet induces alveolar bone resorption similar to that induced by *Aggregatibacter actinomycetemcomitans* infection

**Figure 3:**
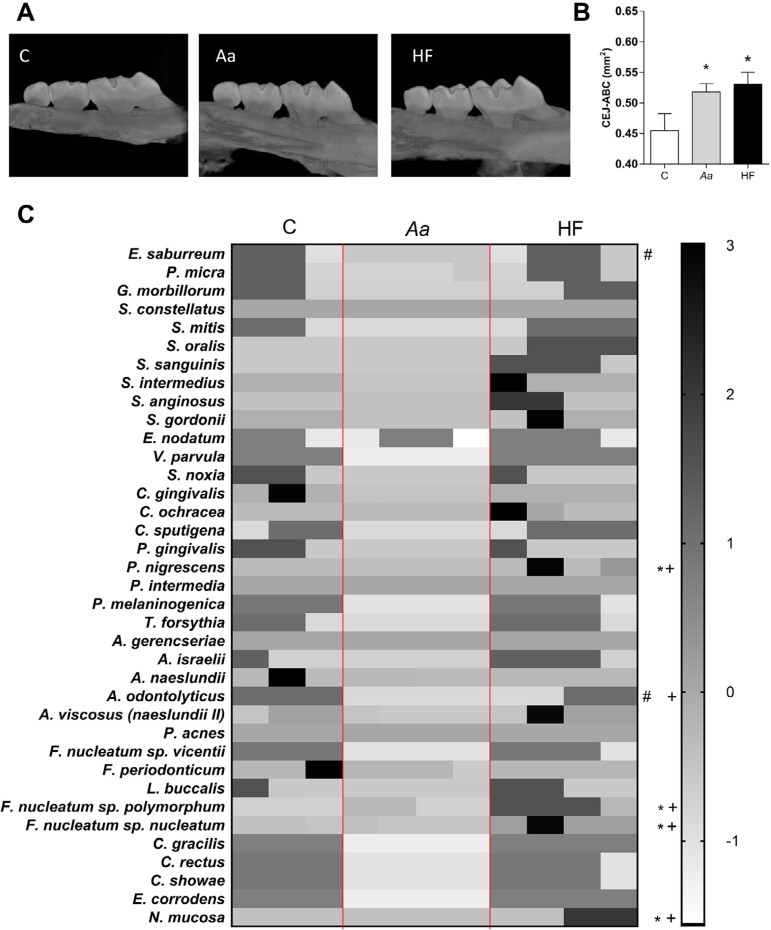
Analysis of periodontal tissues of infected or sham-infected mice subjected to the control or HF diets. (A) Representative images of hemimaxillae of mice from different experimental groups. (B) Analysis of alveolar bone loss after 12 weeks with diet and 4 weeks after oral inoculation of *A. actinomycetemcomitans*. The values (mean ± S.E.M) were obtained from 5 - 7 animals at each point in two independent experiments, with a total of 35 mice. *p<0.05 when compared to control. One-way ANOVA followed by Newman-Keuls. (C) Heat-map representing differences on the oral microbiota analysis between mice fed with the control diet infected or non-infected with *Aa*, and non-infected mice fed with the HF diet. Groups: C — Sham-infected group on the control diet. *Aa* — Group on the control diet, infected with *A. actinomycetemcomitans* HF — Group on the HF diet, non-infected. N=5. *p<0.05 *Aa* compared to HF; #p<0.05 C compared to *Aa*; +p<0.05 C compared to HF. A two-way ANOVA — Bonferroni multiple comparison test was performed. The values (mean ± S.E.M) are representative of two independent experiments

Since *db/db*^-/-^ mice present additional abnormalities,^19^ we used DIO to compare the obesity-induced alveolar bone loss to those induced by *A. actinomycetemcomitans (Aa)*. *Aa* is a bacteria used in a mouse model of PD.^16^ Results show that either the oral inoculation of *Aa* or the use of HF diet induced significant alveolar bone loss when compared to sham-infected mice fed with the control diet (Figures 3A and 3B). The mice fed with the HF diet and infected with *Aa* presented alveolar bone loss similar to those on the HF diet but not infected with *Aa* (Supplementay Figure 1).

### High-fat diet induces oral dysbiosis

Oral microbiota dysbiosis is a crucial feature in the pathogenesis of PD. Thus, the load of bacterial species in the oral microbiota was evaluated by means of DNA-DNA hybridization (Figure 3C). Mice infected with *Aa* presented a lower amount of *Actinomyces odontolyticus* when compared to non-infected mice fed with both types of diet (Figure 3C). Also, mice infected with *Aa* presented lower *Eubacterium saburreum* levels (Figure 3C), when compared to non-infected mice fed with standard diet. Mice fed with the HF diet also presented increased levels of two *Fusobacterium nucleatum* subspecies (*Prevotella nigrescens,* and *Nisseria mucosa*) when compared to the groups fed with the control diet (Figure 3C). Altogether, these results indicate an oral dysbiosis profile associated with HF diet consumption.

### Topical application of chlorhexidine impaired HF diet-induced alveolar bone loss and changes in alveolar bone microarchitecture

**Figure 4:**
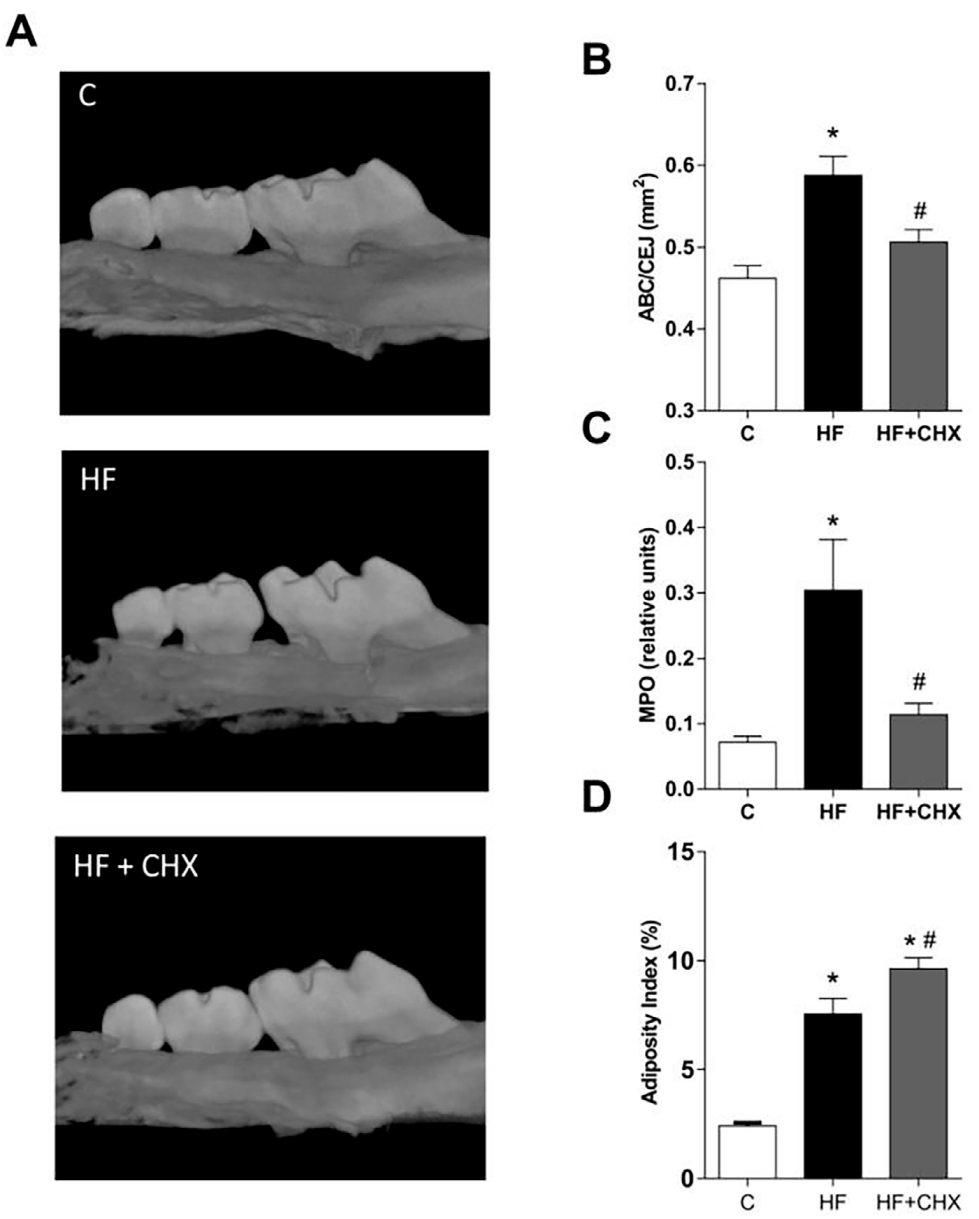
Topical application of CHX impairs HF diet-induced alveolar bone loss. (A) Representative images of hemimaxillae of mice from different experimental groups. (B) Alveolar bone loss evaluation after 12 weeks of diet and 4 weeks after CHX treatment. (C) Neutrophil influx. *p<0.05 when compared to the control group; N=5, #p<0.05 when compared to the HF group. (D) Adiposity measurements after 12 weeks of diet and 4 weeks after CHX treatment.* *p<0.05 when compared to the control group; N=5, #p<0.05 when compared to the HF group. One-way ANOVA followed by Newman-Keuls. The values (mean ± S.E.M) of all experiments were obtained from two independent experiments with a total of 30 mice

We performed an oral topical application of CHX, an antimicrobial agent with a broad anti-microbial action spectrum on both Gram-positive and Gram-negative bacteria. The use of CHX impaired HF diet-induced alveolar bone loss (Figures 4A and 4B). Moreover, the HF diet was associated with increased MPO activity in periodontal tissues, which was also impaired by CHX application (Figure 4C). CHX application did not affect the increased HF diet-induced adiposity (Figure 4D).

**Figure 5:**
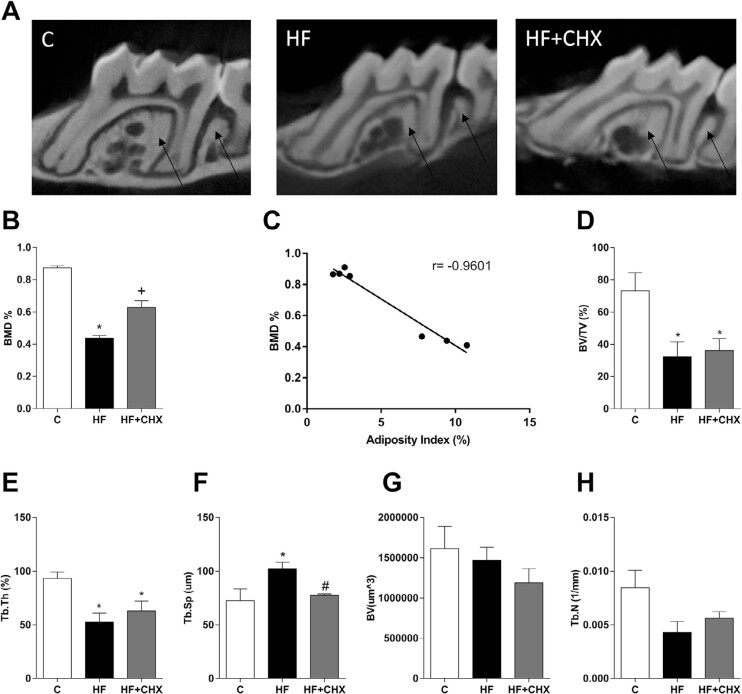
Micro-CT analysis of maxillae of mice fed with the control (C) or HF (HF) diet, or HF diet plus treatment with CHX. (A) Representative Micro-CT images. Arrows indicate the alveolar bone (B) Bone Mineral Density (BMD). (C) Correlation between bone mineral density and adiposity index of the groups C and HF. (D) Bone volume to total volume ratio (BV/TV). (E) Trabecular thickness (Tb.Th). (F) Trabecular spacing (Tb.Sp). (G) Bone volume. (H) Trabecular number (Tb.N). *p<0.05 when compared to the control group. N=4, #p<0.05 when compared to the HF group. +p<0.05 when compared to the Control and HF groups. One-way ANOVA followed by Newman-Keuls. The values (mean ± S.E.M) are representative of two independent experiments

The results of the micro-CT analysis showed that HF diet consumption changed alveolar bone architecture (Figure 5A). HF diet consumption induced a significant reduction in Bone Mineral Density (BMD) when compared to the control diet (Figure 5B). The mice on the HF diet, which received topical application of CHX, showed less bone mineral density loss when compared to the HF diet group (Figure 5B). The results also showed a negative correlation between BMD and the adiposity index in the HF group (r=−0.9601) (Figure 5C). Regardless of the topical application of CHX, the HF diet induced: i) increased percentage ratio of bone volume related to total sample volume (Figure 5D), and ii) decreased trabecular thickness (Figure 5E). On the other hand, the HF diet increased trabecular spacing when compared to mice on the control diet (Figure 5F), which was not observed in mice that received topical application of CHX. There was no significant difference in bone volume (BV mm^3^) (Figure 5G) or in trabecular number (Tb.N 1/mm) (Figure 5H).

## Discussion

The current study aims to relate obesity, changes in microbiota and induction of bone loss, as seen in PD. Experimental studies showed that inflammation exacerbation associates PD and obesity.^20,21^ The results demonstrated that mice given a HF diet or *db/db*^-/-^ presented increased adiposity, increased leptin, and decreased adiponectin serum levels, parameters also observed in humans with obesity.^22,23^ Mice with obesity also presented significant alveolar bone loss and changes in bone microarchitecture when compared to lean mice. Moreover, we observed changes in the oral microbiota of obese mice, characterizing a dysbiotic microbiota along with increased MPO activity in periodontal tissues. Oral topical application of CHX resulted in reversal of alveolar bone loss and bone microarchitecture to levels observed in non-obese mice.

Leptin and adiponectin play significant roles in the host immune response. Leptin has a pro-inflammatory profile, induces production of cytokines in monocytes,^24^ and exerts an impact on bone homeostasis.^25^ The expression of leptin receptors in osteoblasts^26^ and chondrocytes^25^ suggests their direct effects on the bone. In mice, Montalvany-Antonucci, et al.^10^ (2018) showed harmful phenotypes to bone microarchitecture induced by HF diet consumption. Mice fed HF diet had increased expression of inflammatory genes in periodontal ligaments, associated with alveolar bone resorption.^10^ A study showed a significant alveolar bone loss in obese Wistar rats when compared to non-obese rats,^27^ and another showed that HF diet-induced obesity increased the risk of PD.^28^ In the present study, we associated HF diet-induced obesity with significant alveolar bone loss, similar to that induced by *Aa* infection.

Dysbiotic gut microbiota plays a role in the development of obesity.^11,12^ The intake of a HF diet associates with increased number of intestinal Gram-negative bacteria.^29^ Likewise, diet is a determining factor for the maintenance of oral microbiota homeostasis,^30^ and diet changes can result in oral dysbiosis.^31^ For example, consumption of a diet containing 72% of fat and less than 1% of carbohydrate was associated with changes in the subgingival microbiota, with increase of *F. nucleatum* and *P. intermedia*.^32^ Thus, PD and obesity relates to disturbances in the microbiota, with a consensus that pathogenesis of PD has an oral microbiota dysbiosis context.^1,33^ In humans, a study showed that the oral microbial composition differs among subjects with obesity.^34^ Moreover, HF food products reduce efficiency of the immune system, and diminish bactericidal effect on *Porphyromonas gingivalis* in humans.^34^ The current study showed that consumption of an HF diet was associated with oral microbiota dysbiosis. Oral dysbiosis could be a direct effect of a lipid-rich diet on oral microbiota composition, resulting in inflammation and alveolar bone loss; however, *db/db*^-/-^ mice, given that a standard diet presented similar alveolar bone loss and increased MPO activity when compared to WT mice, showing that both phenotypes were an indirect effect of obesity.

The analysis of maxillae of *db/db*^-/-^ mice also showed decreased levels of resistin, an important adipokine with a regulatory role in insulin resistance and inflammation.^36^ A study investigated the associations between insulin resistance and periodontitis in non-abdominally patients with obesity and concluded that insulin resistance can be considered an independent risk factor of PD.^37^ In patients with osteoporosis, resistin was also negatively correlated with BMD of long bones.^38^ Chemerin is an adipokine with an important role in the activation and differentiation of osteoclasts, associated with obesity-induced alveolar bone loss.^39^ Herein, the *db/db*^-/-^ mice presented increased chemerin levels both in serum and in periodontal tissues. These findings suggest a possible role of adipokines in bones of obese mice, as we observed changes in adipokines levels both in serum and in periodontal tissues.

Since PD is associated with changes in oral microbiota^1^, we analyzed dysbiotic bacteria in the oral cavity of our obese mice. The analysis showed that the HF diet increased *F. nucleatum*, *P. nigrescens* and *N. mucosa* counts when compared to the control group. However, the limitations of the Checkerboard DNA-DNA hybridization technique need to be taken into consideration when interpreting these data. A study showed the association of *F. nucleatum* with the pathogenesis of PD, facilitating the colonization of bacteria associated with PD.^1^ In the dysbiosis context, *P. nigrescens* changes the expression of virulence factors, becoming a putative pathogen, without changes in its abundance in oral biofilm.^40^

By observing dysbiosis in the oral microbiota of obese mice, we evaluated whether decreasing local bacterial load with CHX could reverse the changes caused by obesity on the alveolar bone. Topical application of CHX in mice on HF diet was associated with an increased adiposity index but impaired alveolar bone loss and resulted in less neutrophil infiltrate in periodontal tissues. Moreover, bone architecture improved when compared to mice on the HF diet without CHX application. Despite the increased adiposity index in CHX group, probably due to changes in intestinal microbiota, these results demonstrate a role for dysbiotic oral microbiota in breakdown of periodontal tissues and changes in bone microarchitecture induced by the HF diet, as well as strengthening the impact of neutrophils on alveolar bone loss associated with oral dysbiosis.

Neutrophils present dual effect in the pathogenesis of PD: the protective role involves the maintenance of tissue homeostasis and the destructive role involves the inflammatory bone loss.^33^ Neutrophils are involved in activation and differentiation of osteoclasts, multinucleated cells involved in bone resorption, through the RANKL membrane.^41^ Obesity models resulted in increased MPO activity in periodontal tissues, suggesting that neutrophils may exert an impact on obesity-induced alveolar bone loss. Future research studies are necessary to elucidate this hypothesis.

Although our study shows a clear association between obesity, dysbiosis, periodontal bone loss, and inflammation, it presents certain limitations. For example, a deep sequencing analysis of the oral microbiota is suitable to obtain a more in-depth profile of the oral microbiota composition, mainly during dysbiosis. Moreover, despite the use of *in vivo* studies, including gene knockout mice, suitable for detailed mechanisms, we did not sufficiently evaluate some inflammatory markers such as cytokines in this study.

## Conclusion

HF diet-induced oral dysbiosis and subsequent alveolar bone loss. CHX use impaired bone changes and neutrophil influx but had no effect on adiposity gain. Thus, the dysbiotic oral microbiota plays an important role in obesity-induced alveolar bone loss.

## Figures and Tables

**Supplementary figure 1:**
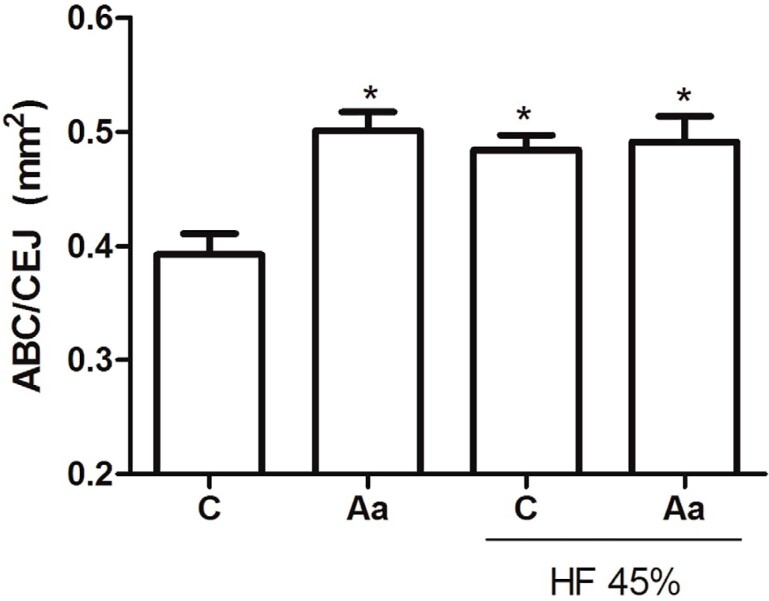
Analysis of alveolar bone loss of infected (Aa) or sham-infected (C) mice submitted to standard or HF 45% diet. Analysis of alveolar bone loss after 12 weeks with control or HF 45% diet, and 4 weeks after oral inoculation of *A. actinomycetemcomitans* (Aa). The values (means ± S.E.M) were obtained from 5 mice at each point in one independent experiment. *p<0.05 when compared to control. ANOVA followed by Newman-Keuls
